# Lessons Learned about the Design and Active Characterization of On-Body Antennas in the 2.4 GHz Frequency Band

**DOI:** 10.3390/s20010224

**Published:** 2019-12-31

**Authors:** David Naranjo-Hernández, Javier Reina-Tosina, Laura M. Roa

**Affiliations:** Biomedical Engineering Group, University of Seville, 41092 Seville, Spain; jreina@us.es (J.R.-T.); lroa@us.es (L.M.R.)

**Keywords:** on-body antenna, Body Sensor Network, impedance matching, anechoic chamber, radiation pattern

## Abstract

This work addresses the design and experimental characterization of on-body antennas, which play an essential role within Body Sensor Networks. Four antenna designs were selected from a set of eighteen antenna choices and finally implemented for both passive and active measurements. The issues raised during the process of this work (requirements study, technology selection, development and optimization of antennas, impedance matching, unbalanced to balanced transformation, passive and active characterization, off-body and on-body configurations, etc.) were studied and solved, driving a methodology for the characterization of on-body antennas, including transceiver effects. Despite the influence of the body, the antennas showed appropriate results for an in-door environment. Another novelty is the proposal and validation of a phantom to emulate human experimentation. The differences between experimental and simulated results highlight a set of circumstances to be taken into account during the design process of an on-body antenna: more comprehensive simulation schemes to take into account the hardware effects and a custom design process that considers the application for which the device will be used, as well as the effects that can be caused by the human body.

## 1. Introduction

Body sensor networks (BSN) are a promising solution to the challenges of personalized healthcare [1,2,3,4,5,6]. These body-centric communications are mainly developed in the 2.4 GHz frequency band using low-power technologies such as Bluetooth Low Energy (BLE) [7,8] or IEEE 802.15.4 [9,10,11,12,13] and IEEE 802.15.6 [14,15,16,17,18] standards. A critical element in BSN are the antennas [8,19,20,21,22] and their design specifications are especially crucial in the case of biomedical applications, in which small ultra-light devices, which suppose no hindrance on a subject’s daily life, are required. It is also desirable that they have a minimum consumption to reduce the need for battery recharge or change, and immunity against external interferences to safeguard the privacy of communications [4]. Other reasons include the need for efficient and secure bidirectional communication links, which allow the reprogramming and personalization of devices [3,4,5]. Although it is evident that during the last years important advances about the design and development of embedded antennas for micro-sized devices, even those with the capacity of in-body integration, have been achieved [20,23,24], both the aforementioned restrictions and design challenges, as well as the trade-off between the size of the antenna and its efficiency [25], highlight the need for advancing in research concerning portable technologies that permit the personalization and adaptation of communication systems in general, and in particular of antennas.

On the other hand, it must be considered that in BSN the antennas are arranged in devices located close to or in contact with the human body, a circumstance that may affect the performance of the antenna [22,26,27], which makes the recalculation of parameters necessary, in detriment of their efficiency, thus causing a trade-off to be made in each case. The characterization of BSN antennas is normally addressed without taking into account the effects of the transceiver itself and the consideration of a finite ground plane, which can greatly influence the antenna performance [28]. All of these, combined with the fact that the propagation mechanisms through the human body are not unknown in enough detail, underline the need for more exhaustive studies that analyze the influence of the human body on antennas and viceversa, to be undertaken [29].

The passive measurement inside an anechoic chamber is the standard method for the experimental characterization of antennas [8,21,27,29,30]. However, this method may be unsuitable if the characterization of the antenna integrated in a self-powered electronic device is required, given that the hardware components also influence the radiation characteristics [23,31]. In addition, it is possible that the antenna is matched to an impedance different from 50 ohms. In these cases it is common to use baluns, which can also influence the antenna results [32]. To the best of the authors’ knowledge, there is no standardized methodology for the experimental characterization of antennas integrated in self-powered electronic devices in the context of the BSN.

In this sense, this paper describes the issues encountered during the design and characterization process of a set of on-body antennas in anechoic chamber, and the solutions used to solve the problems found. This paper extends the work presented in [33] with the addition of three different antennas and a more detailed description of the requirements analysis, design, development and characterization procedures. Other extensions are a rational procedure for active measurements of BSN antennas integrated with the transceiver in battery-powered devices in order to obtain more realistic results, an analysis of the influence of the human body by means of on-body experimentation inside anechoic chamber and the proposal and validation of a phantom to emulate and facilitate human experimentation with antennas.

## 2. Materials and Methods

### 2.1. Issues Related to the Biomedical Application

The antenna will be integrated into a smart accelerometer sensor device called SoM (Sensor of Movement). This sensor is part of a low-cost e-Health system for fall detection [34]. The SoM is integrated in a biocompatible waterproof skin patch that is comfortably fixed on the back at the height of the sacrum for monitoring 24 h a day [34]. These circumstances impose to the antenna a minimum size, cost and weight, and as the device must have a long autonomy, energy efficiency is another major requirement.

The SoM is connected wirelessly with a device called DAD (Decision-Analysis Device), and this, in turn, with a remote healthcare center. The DAD is also carried by the person being monitored, although it can also be found in another position within the coverage area, either on an eventual or permanent basis. In this sense, the radiation pattern should be omnidirectional as far as possible.

Furthermore, as the wireless transmission is the major responsible for energy consumption in BSNs [4], information processing is distributed among the SoM and DAD to summarize and abstract the information transmitted with the consequent reduction of energy expenditure in communications [35].

To reduce the energy consumption in communications, only data are sent when an event susceptible of being considered a fall is detected [36]. In this way, low data rate is suitable for the communications link, and to support the personalization and adaptation of the SoM software, an efficient bidirectional link is required.

### 2.2. Issues Related to the Communication Technology Selection

Considering the needs established for the application (energy efficiency, low data rate, bidirectional link, etc.), IEEE 802.15.4 standard was chosen as the underlying communications technology [36] and Texas Instruments CC2431 transceiver was selected considering its low energy consumption and low cost [37]. According to the manufacturer’s specifications, new antenna requirements were established to ensure efficient and robust communications: input impedance close to 60+j164 Ω; the antenna should be balanced, or a balun should be added otherwise [37]; minimum return loss above 10 dB; bandwidth should safeguard the frequency spectrum between 2.403 and 2.482 GHz; gain such that a range of 5 m is guaranteed in communications. As low power is used, safety conditions are established (ANSI C95.1, ICNIRP) [38]. Given these restrictions, together with the fact that IEEE 802.15.4 works in the range of 2.4 GHz, printed circuit board (PCB) microstrip implementation was selected, against other alternatives such as chip or wired antennas, due to its low cost, simple implementation, high performance and small size in such frequency range.

### 2.3. Issues Related to the Design and Simulation Process

The preliminary conditions related to the antenna design were the following:

- A previously defined hardware design for the application case (fall detection): accelerometer, microcontroller and auxiliary components.
- The transceiver to be used is the CC2431 from Texas Instruments.
- Microstrip technology selected for the antenna design.
- For the manufacture of the PCB, FR4 Epoxy substrate has been selected because it is a low-cost material, with high dielectric constant, acceptable loss characteristics, reduced weight, providing compact radiation structure [39,40,41].
- The thickness of the FR4 substrate was set at 1.6 mm to make the board rigid enough for mounting the devices, provide mechanical robustness and reliability, in addition, since it is a commercially available standard thickness, commonly used for the manufacture of microstrip antennas [42,43].
- The hardware defines a location of components, a top layer and a ground plane whose dimensions and size are optimized, which will not be modified during the antenna design process, so that the antenna will be incorporated into the previous hardware design.

The consideration of the electronics associated with the sensor device places additional requirements which makes difficult a single choice without testing the implications of transceiver, finite ground plane effects, sensor itself and body effects on performance. For that reason, several designs were considered. The particular choices were an attempt to balance the use of standard antenna design alternatives with the recommendations of the transceiver’s manufacturer.

Ansys HFSS software, based on electromagnetic finite-element-method (FEM), was used to design, optimize and simulate the radiation characteristics of the antennas (gain, return loss, radiation pattern, etc.). To obtain consistent simulation results, the output port impedance was renormalized to match the optimum load impedance of the transceiver.

The uncertainty about the implication of these preliminary conditions on overall performance (on-body operation, finite ground-plane effects, companion sensor electronics, etc.), suggested the consideration of several starting antenna designs. The particular choices balanced the use of standard topologies with the transceiver manufacturer recommendations:

- Three microstrip antennas recommended by the transceiver manufacturer: Folded dipole, Meandered Inverted F Antenna (MIFA) and Inverted F Antenna (IFA) [44,45,46].
- Seven microstrip antennas proposed in the literature for biomedical applications [23,47,48,49,50,51].
- Eight designs derived from generic antennas: two types of monopole, three types of dipole, three types of circular antennas around the hardware, and a yagi antenna adapted to the microstrip technology [52].

**Figure 1:**
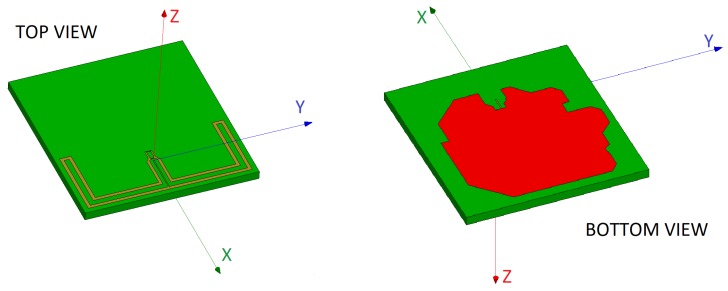
Example of an antenna layout considered for design.

The antenna design and optimization process was carried out as follows:

- **Starting design of the antennas**: All the antennas were implemented in the electromagnetic simulation software through different design tests for adaptation to a basic implementation of the sensor hardware that considered the dimensions of the ground plane and the thickness of the substrate. Figure 1 shows as an example an implementation of one of the analyzed antennas.
- **Selection of sensitive dimensions**: Through an analysis process, the dimensions that allowed the adjustment of the resonant frequency and bandwidth were selected.
- **Iterative optimization process of the antennas**: The radiation characteristics of each of the designs were optimized through an iterative adjustment of sizes, positions and values of the discrete impedance matching components (resistors, inductors, and capacitors). All the antennas were tuned by modifying the adjustable parameters of the antennas to meet the preliminary conditions related to the antenna design and to robustly comply with the design requirements established in the Section 2.2, maintaining a trade-off with the minimization of the device size. In general, the dimensions were changed iteratively to get, if possible, a resonant frequency centered around 2.45 GHz and return losses greater than 10 dB in the frequency range between 2.4 and 2.5 GHz.
- **Sensitivity analysis of the antennas**: On the selected dimensions a sensitivity analysis was performed to rule out those configurations very sensitive to small size changes.
- **Evaluation of the radiation pattern** of the antennas to select those configurations that provided a trade-off between the gain and an omnidirectional radiation pattern.

**Figure 2:**
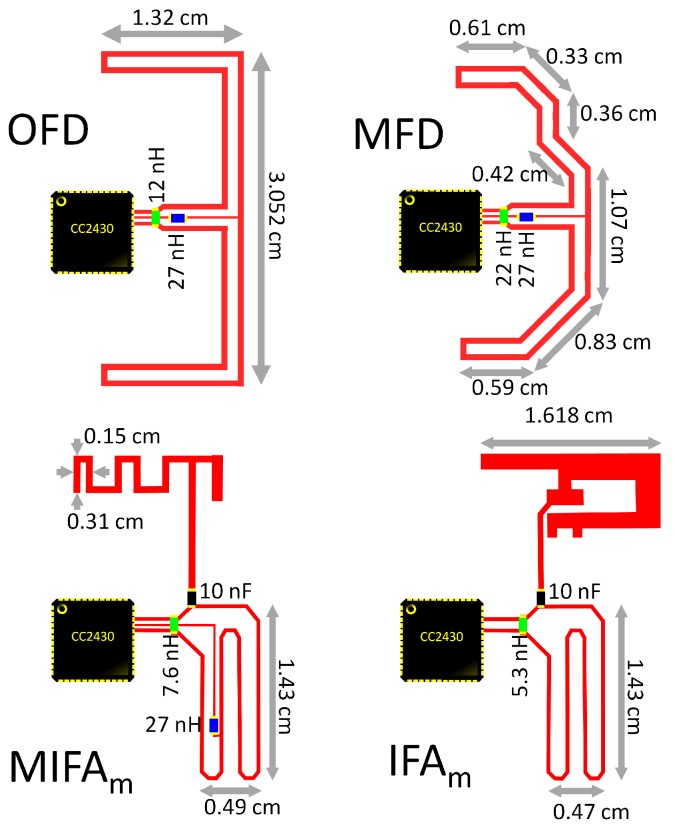
Layout of the selected antennas.

Table 1 summarizes the main results of the antennas under test, both the original and the adapted antennas after the iterative optimization process when this was possible. According to the simulations, the final four antenna designs showing best performance were selected for implementation (see Figure 2):

- **Orthogonal Folded Dipole (OFD)**: A folded dipole with balanced output, but with an orthogonal shape in order to be adapted to the circuit size, thus reducing the overall size of the device. The use of a balun is not necessary since OFD is already a balanced antenna.
- **Matched Folded Dipole (MFD)**: A modification of a folded dipole in order to follow to the shape of the device, thus reducing the overall size.
- **Modified MIFA** (MIFA_m_): Despite its small size, this antenna is not balanced. Therefore, an additional balun was designed according to the characteristics of both antenna and sensor device.
- **Modified IFA** (IFA_m_): It requires a larger surface and needs to be matched with a balun too.

Figure 2 shows in detail the dimensions of these antennas (undescribed sizes are equivalent to those shown in [44,45,46]).

### 2.4. Issues Related to the Anechoic Chamber Measurements

**Figure 3:**
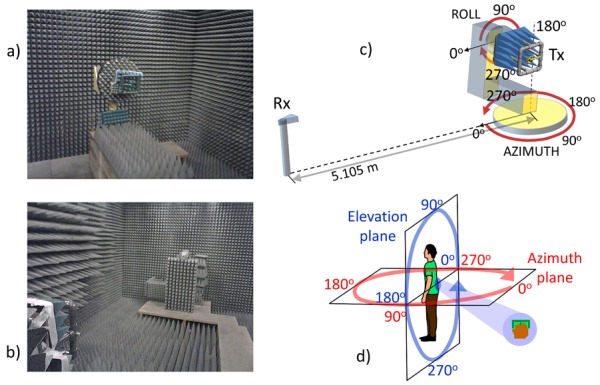
(**a**) Interior of the anechoic chamber with the Tx in the background; (**b**) Interior of the anechoic chamber with the Rx in the background; (**c**) Scheme of the arrangement of the Tx and Rx devices inside the anechoic chamber and definition of the measurement planes; (**d**) Distribution of angles for the measurements in elevation and azimuth planes inside the anechoic chamber.

**Figure 4:**
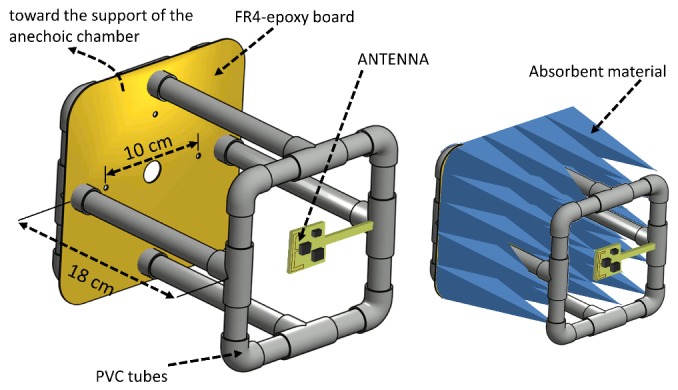
Non-conductive PVC support for anechoic chamber measurements.

The experimental characterization of the antennas was carried out inside an electromagnetic anechoic chamber. Figure 3 shows two pictures of the interior of the anechoic chamber. To avoid the electromagnetic compatibility problems caused by the metallic support of the transmitter (Tx) antenna, a non-conductive PVC (polyvinyl chloride) support was manufactured, since this material has dielectric properties similar to air. This solution has already been used in other works because of its low cost and because it allows to manufacture very robust structures by assembling plumbing pipes and joints [53,54,55,56]. The effects of this type of support have been considered negligible in some studies [57]. The internal space of the support and the metallic components were covered by the same electromagnetic radiation attenuation absorbers as those used on the anechoic chamber walls. Although the effects of the metal support cannot be completely removed, it is a suitable solution to approach a free space situation [56]. Figure 4 shows a diagram of the non-conductive support and the arrangement of the Tx antenna on it.

**Figure 5:**
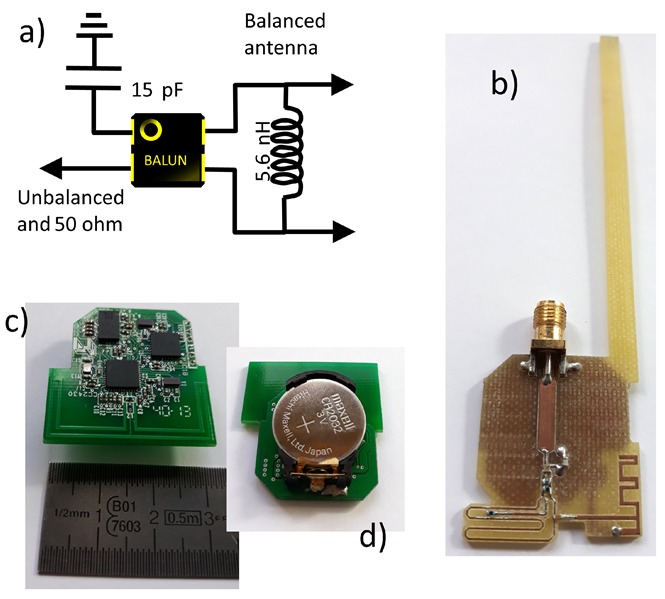
(**a**) Impedance network and balun for P1 prototypes; (**b**) Example of implementation of a prototype P1 of the MIFA_m_ antenna; (**c**) Example of implementation of a prototype P2 of the OFD antenna (TOP VIEW); (**d**) BOTTOM VIEW.

According to transceiver restrictions, the antennas are balanced and with input impedance close to 60+j164 Ω. As the measurement equipment (HP8510-B automatic vector network analyzer of HP) input was unbalanced, a impedance network and a balun [58] were included in a first set of prototypes (P1) to perform the single-ended to differential conversion, and match the antenna impedance to the measurement equipment (see Figure 5).

However, the received signal from the passive prototypes was very weak, possibly due to losses in the impedance matching network. To solve this problem, and although another type of balun or impedance matching network could have been used to feed the antennas from network analyzer of the anechoic chamber, it was decided to go one step further and propose a different solution to the standard method based on passive measurements. Instead, antenna performance was evaluated in their context of use, including all the hardware associated with the portable device in which the antenna would be located. In this case, the transceiver itself is the one that generates the antenna’s power signal, which is powered by the battery of the portable device. This method proposes to be a more realistic approach to the conditions of use of the antenna, incorporating the external elements and effects that can influence the operation of the antennas.

To address this issue, four complete SoM prototypes (P2) were implemented, each of them with its own battery supply and one of the selected antennas connected to the transceiver (see Figure 5). The advantage of this configuration is that the effects derived from the hardware of the device (shape, dimensions, electronic and metallic components, properties of the PCB, etc.) are integrated into the experimental characterization, but also remove the effects caused by the long cables that connect the Tx antenna to the measuring equipment. A lithium button cell model CR2032 was used to power the prototypes. This battery was located at the bottom of the PCB on a battery holder. The location of the battery is shown in Figure 5. Details about the consumption of the device were commented in [36].

Since the transceiver could not be configured to transmit continuously (the communications protocol requires the use of listening periods), a duty cycle of 81.72% was achieved by activating sequentially and successively the transmission and reception of data for 5.472 ms and 1.224 ms, respectively. The selected transmission frequency was 2.4448 GHz, since it is located at an intermediate point of the transmission range. However, a stable measurement of the network analyzer was not possible due to the continuous phase changes that occur in the carrier as a result of the use of the IEEE 802.15.4 standard protocol. To solve this issue, a FSL8 spectrum analyzer of Rohde & Schwarz was used.

Figure 3 shows the arrangement of the measurement elements inside the anechoic chamber, as well as the definition of the planes (azimuth and elevation) used to characterize the radiation diagrams of the antennas taking as reference the sensor position described in Section 2.1.

The azimuth plane pattern was obtained setting the roll angle to 90° and measuring at different azimuth angles with a separation of 1°. The correspondence between angles was set considering the schemes shown in Figure 3. The spectrum analyzer was configured with zero span option at the Tx frequency and the power level received was averaged to assume the duty cycle discontinuity by setting a sweep time of 80 s for 501 samples. Three measurements were performed for each of the positions in different time instants. A larger number of measurements was not considered due to the low variability and the average was considered as the final value. When any element of the support could obstruct the direct sight path between the Tx and the receiver (Rx), a specular position of the metal support on the azimuth rotation axis was established by modifying the azimuth and roll angles but keeping the antenna in a configuration equivalent to the initial position. The elevation plane pattern was obtained setting the azimuth angle to 270° and measuring at different roll angles in increments of 1°. In the same way, three measurements were taken for each orientation. This procedure was repeated for each of the antennas.

The antennas were characterized in four different configurations for a more comprehensive evaluation:

- **Off-body**: This condition was emulated following the procedure described above using the non-conductive PVC support and electromagnetic radiation attenuation absorbers inside the support as Figure 4 shows.
- **Phantom to emulate on-body condition (separated)**: In this case, the space between the antenna and the metal support was occupied by a phantom of biological material to emulate the presence of the human body. Containers of liquids or gels that simulate the electrical properties of the body tissues have been used [29], but in this work a biological material of similar structure (skin, fat and muscle) is proposed. A chicken for food consumption was selected to implement the phantom (2.45 kg, clean inside, featherless at room temperature), which was fixed to the support. The phantom was covered by a very thin film (15 μm) of non-conductive material (polyethylene and polypropylene) to prevent electrical conduction effects. The antenna was arranged to maintain a separation of 0.5 cm from the surface of the phantom and located over the center of the area corresponding to the breast.
- **Phantom to emulate on-body condition (fixed)**: Equivalent to the previous condition, but in this case the sensor was arranged in direct contact with the surface of the phantom. The side of the PCB (FR4 epoxi) in contact with the phantom surface was free of copper, and the antenna was located on the opposite side of the PCB.
- **On-body**: In this case, the metallic support was discarded, placing on its position a volunteer (male, 42 years of age, height of 1.73 cm and weight of 82 kg). Each of the P2 prototypes were placed on the volunteer according to Section 2.1. In each experiment, the volunteer oriented himself inside the anechoic chamber towards a fixed azimuth angle using a system of visual references. The average value of thirty measurements was recorded. A greater number of measurements was necessary in this case to estimate the mean value due to the increase in variability as a consequence of performing measurements on a person. Different experiments were carried out to go through the azimuthal plane in increments of 15°. In order to obtain the elevation plane pattern, the volunteer was oriented in a direction transverse to the line connecting Tx with Rx, manually varying the orientation angle of the sensor on the volunteer’s back in increments of 15° in each of the experiments.

## 3. Results

### 3.1. Simulation Results

**Figure 6:**
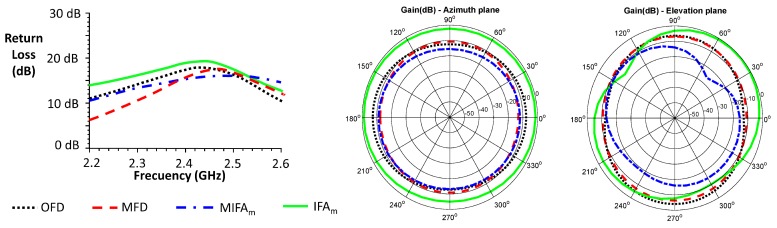
Simulated return losses and radiation pattern (azimuth and elevation planes) of the proposed antennas.

Figure 6 shows some of the simulation results of the implemented antennas. OFD antenna has an omnidirectional radiation pattern in the normal plane to the antenna. It meets the return loss specification over all the frequency range and provides a very high bandwidth, around 400 MHz. MFD bandwidth decreases compared with that presented by the OFD. MIFA_m_ and IFA_m_ antennas robustly meet the return loss profile and they also present a large bandwidth. The gain of all the antennas is suitable for a BSN application (5-m communication range guaranteed) and their radiation pattern is omnidirectional in the azimuth plane. On the other hand, the high bandwidth obtained in all the cases makes them robust against possible mismatches due to a poor repeatability in the PCB implementation.

### 3.2. Anechoic Chamber Results

**Figure 7:**
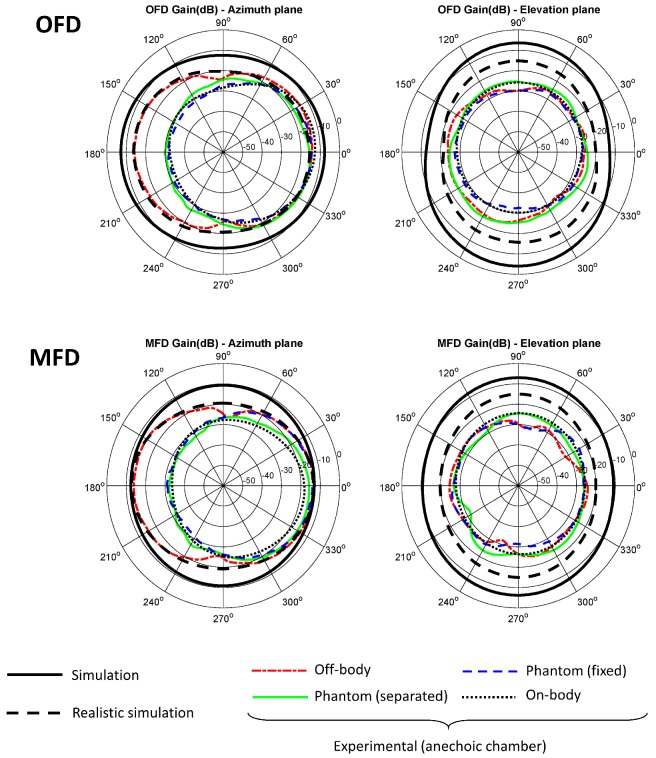
Simulated and experimental radiation patterns in azimuth and elevation planes of OFD and MFD antennas.

**Figure 8:**
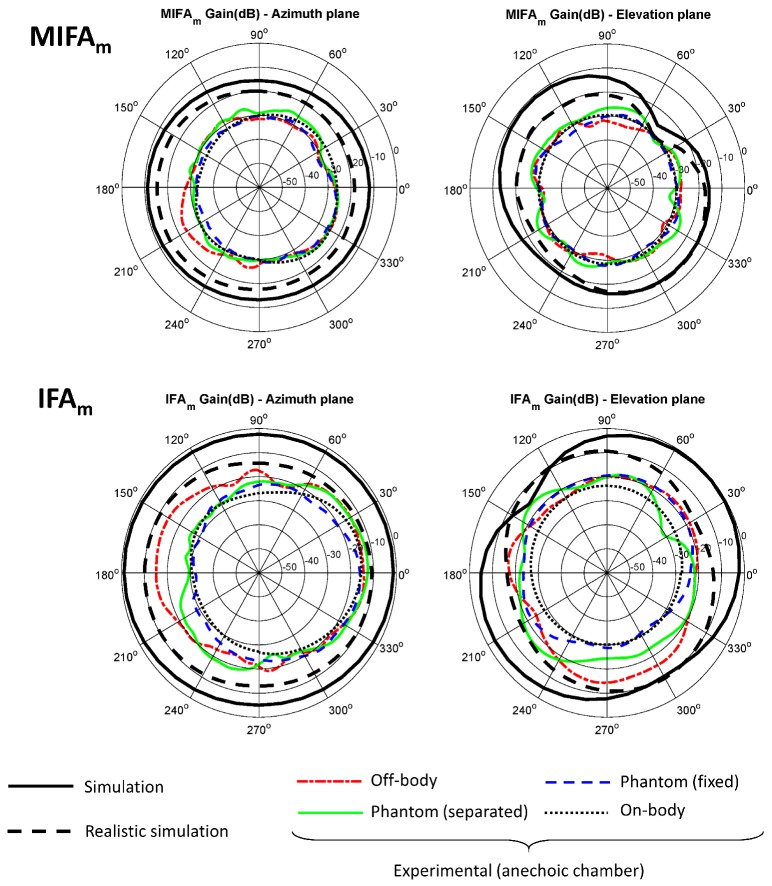
Simulated and experimental radiation patterns in azimuth and elevation planes of MIFA_m_ and IFA_m_ antennas.

The gain of the Tx antenna was estimated using Friis Transmission Equation from the average received power. For the calculations, a transmitted power of 0 dBm (value set in the CC2431 transceiver) was considered, a Rx antenna gain of 8.575 dB (LB-20245 of AINFO Inc., according to the manufacturer’s specifications at 2.4448 GHz), a transmission distance of 5.105 m and a correction factor of 1.224 to take into account the attenuation caused by the duty cycle. Both azimuth and elevation planes patterns are shown in Figure 7 and Figure 8 for the different evaluations inside the anechoic chamber and referenced over the simulated results.

In order to allow a comparison between the simulation and the experimental results, the different radiation patterns are shown together. The radiation patterns in the elevation and azimuth planes are shown according to the angles and orientations described in Figure 3. The simulation results have been obtained using the HFSS software and the experimental results inside the anechoic chamber. Simulation results are shown in thick solid line and black color using the basic model described in the Section 2.3 that included the antenna, the substrate and the ground plane. A narrow dotted line in red color shows the results of the off-body experiment inside the anechoic chamber using the non-conductive PVC support and attenuation prisms to emulate a free space condition. The narrow solid line in green color corresponds to the experiments with the biological phantom inside the PVC support and the antenna 0.5 cm apart from it. The narrow dashed line in blue color shows the results with the biological phantom, but in this case without any separation. The radiation patterns of the antenna in a realistic situation on a person’s body is described in narrow dotted line and black color.

**Figure 9:**
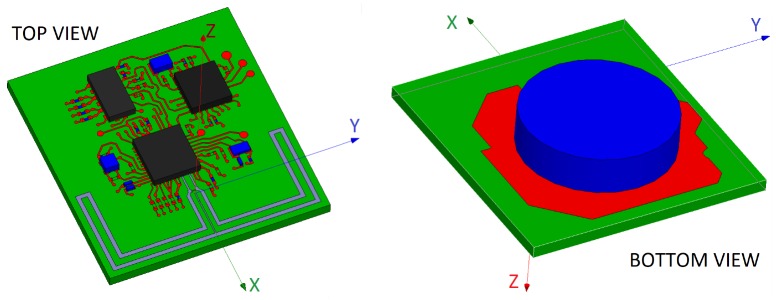
Example of an antenna design employed for realistic simulations. Note: The color of the components is for visual purposes only, and not indicative of the material.

Given the disagreements between the simulation and experimental results, it was considered convenient to repeat the simulations including in the models the metallic elements that make up the tracks and pads of the sensor hardware, as well as the battery and other dielectric and conductive elements related to the electronic components. Figure 9 shows by way of example the realistic representation of the model related to one of the antennas (OFD). Such simulation is superimposed on the radiation diagrams of Figure 7 and Figure 8 as a thick dashed line in black color.

## 4. Discussion

A mismatch between the experimental results and simulations can be observed in Figure 7 and Figure 8. Probable causes of the differences may be the losses attributable to the elements not considered in the design and simulation phase: uncertainties due to the manufacturing process, calibration or measurement errors, finite ground plane effects, influence of the metallic and electronic components of the device’s complete hardware, properties of the transceiver and the communications protocol, but also other losses derived from the front-end of the measurement equipment, such as possible signal losses by impedance mismatch in the Tx or Rx antennas, in the coaxial cable that connects them to the spectrum analyzer or in the connectors [59,60,61,62,63,64]. It was not possible to perform any type of calibration to remove the losses associated with the transceiver interfaces, since it was not possible to establish any reference in the received signal if the battery-powered transceiver was used as transmitter.

However, this work contributed a methodology for the evaluation of the performance of antennas integrated in the complete hardware of a portable device, including the battery. This proposed procedure differs from the standard method for the passive evaluation of antennas inside an anechoic chamber using a network analyzer with antennas matched to 50-ohm. Under normal circumstances, the standard method provides an effective way to evaluate antenna performance, with good correspondence between the simulation results and the experimental results. However, sometimes these differences can be important as a result of the loss of control in the elements that influence the antennas or external effects [60,61,62,63,64,65,66]. The proposed method, despite its higher complexity, assumes many of the effects that can influence the performance of the antennas, so it can represent a better approach to reality.

Although the simulation with basic elements of the antenna and the ground plane reported differences with respect to the experimental results in all the cases analyzed, similar results have been found in other studies with antennas for personal communications [67,68]. To address this issue, a more realistic simulation was performed including explicitly the main hardware elements that can affect the radiation characteristics of the antenna (metallic elements of the PCB, battery, etc.). The realistic simulation was closer to the experimental results in all the cases. In the particular case of OFD and MFD antennas, realistic simulation largely coincided with the results in off-body experiments. On the other hand, in the elevation plane, the differences, although minor, were around 10 dB. A possible cause of these discrepancies may be related to losses due to the polarization of the antennas. Greater differences between the realistic and experimental simulation were found in the cases of the MIFA_m_ and IFA_m_ antennas, which can be due to the greater complexity of these antennas, and therefore to the increase in the number of elements that may affect their performance. It should be remembered that both antennas include a balun.

The results obtained with the proposed phantom (a featherless chicken for food consumption) were qualitatively and quantitatively equivalent to those obtained in on-body experimentation. The main advantage offered by this phantom is that it allows the use of the automatic positioning system of the anechoic chamber (roll and azimuth). This would favor the agility of experimentation and the possibility of tackling in a simple way orientations and positions difficult to evaluate in human experimentation.

If the results obtained with the phantom are analyzed, a configuration in which the sensor is separated from the surface of the body is slightly beneficial with respect to another configuration in which the sensor is fixed on the surface thereof.

Despite the influence of the body, the four antennas showed a fairly omnidirectional behavior, which is beneficial for the use application of the sensor. In addition, although there are important differences regarding the simulation, the experimental results are considered sufficient for an in-door environment. The worst performances were obtained for the MIFA_m_ antenna, but the other antennas showed similar behavior.

## 5. Conclusions

This work has analyzed the issues raised during the design, development and characterization processes of a set of on-body antennas. The first point was to analyze the needs derived from the application where the antennas will be used. The second point was to address the communication technology issues (selection of the communication link technology, new requirements associated, antenna type selection, etc.). Eighteen different antenna designs were performed and optimized using an electromagnetic simulation software through an iterative adjustment of sizes, positions and impedance matching components. The final four antenna designs showing best performance in simulation were selected for implementation and experimental characterization.

The common method for antenna characterization, passive antenna measurement in an anechoic chamber, was discarded due to the weak levels of the received signal. The cause of this problem was possibly the losses in the impedance matching network used to match the antennas to the measuring equipment: single-ended to differential conversion; 50-Ohm measurement equipment impedance matching.

To deal with these issues, active characterization of the antennas was performed following a specific procedure conceived for the analysis of assembled antennas in self-powered devices. This method enables the evaluation of the antenna performance in conditions close to reality, including the effects caused by the hardware (the shape and size of the metal and dielectric elements of the circuit, the influence of electronic components or the effects derived of the transceiver and the communications protocol, among others), but also removing unwanted effects derived from the Tx front-end of the measurement equipment, such as possible signal losses in the coaxial cable or caused by impedance mismatch in the antenna and connectors. The problems caused by the communications protocol were solved with the use of a spectrum analyzer instead of a network analyzer. The effects caused by the human body in the context of a BSN were analyzed through on-body experimentation. In addition, it has been proposed and validated the use of a phantom to emulate the on-body experimentation inside an anechoic chamber, suitable for a rapid preliminary assessment unconstrained by the limitations that the automatic positioning and orientation of the human body would have.

A realistic simulation of the elements that make up the hardware of self-powered devices made it possible to verify that the battery and the metallic elements close to the antenna affect its performance. The experimental results were closer to the simulation results when the conductive elements that make up the PCB hardware are explicitly included in the simulations. Generic electromagnetic simulation tools are not prepared to model and simulate these components close to the antennas. It is advisable to develop new and more comprehensive design and simulation schemes that allow incorporating the effects of the complete hardware of the device in which the antenna would be integrated; the presence of the human body and its effects should be incorporated into a custom design process, adapted to the specific application of use; and, to minimize possible deviations from the design requirements, it is advisable to incorporate a cycle of iterative spiral development with intermediate experimental evaluation stages.

## Figures and Tables

**Table 1:** Antenna selection table based on compliance with design requirements.

Antenna	Type	Resonant Frequency	Bandwidth	Gain	Omnidirectionality
Folded dipole [44]	Original	✔	-	-	✔
	Adaptation 1 (OFD)	✔	✔	✔	✔
	Adaptation 2 (MFD)	✔	✔	✔	✔
	Adaptation 3	✔	-	-	✔
MIFA [45]	Original	-	-	-	✔
	Adapted (MIFA_m_)	✔	✔	✔	✔
IFA [46]	Original	✔	-	-	✔
	Adapted (IFA_m_)	✔	✔	✔	✔
[47]	Original	-	-	-	✔
	Adapted	✔	-	-	✔
[23]	Original	-	-	-	-
	Original	✔	✔	-	-
[48]	Original	-	-	-	-
	Adapted	✔	-	-	-
[49]	Original	-	-	-	-
	Original	✔	-	-	-
[50]	Original	✔	-	-	-
-	Adapted	✔	✔	-	✔
[51]	Original	✔	-	-	-
	Adapted	✔	-	-	✔
Monopole 1	Adapted	✔	-	-	✔
Monopole 2	Adapted	✔	-	-	✔
Dipole 1	Adapted	✔	-	-	✔
Dipole 2	Adapted	✔	✔	-	✔
Dipole 3	Adapted	✔	✔	-	✔
Circular 1	Adapted	✔	-	-	✔
Circular 2	Adapted	-	-	-	-
Circular 3	Adapted	-	-	-	✔
Yagi	Adapted	✔	-	-	-

## Abbreviations

The following abbreviations are used in this manuscript:

BLE	Bluetooth Low Energy
BSN	Body sensor network
DAD	Decision-Analysis Device
FEM	Finite-element-method
IFA	Inverted F antenna
IFA_m_	Modified IFA
MFD	Matched folded dipole
MIFA	Meandered inverted F antenna
MIFA_m_	Modified MIFA
OFD	Orthogonal folded dipole
PCB	printed circuit board
PVC	Polyvinyl chloride
P1	First set of prototypes
P2	Complete SoM prototypes
Rx	Receiver
SoM	Sensor of Movement
Tx	Transmitter
